# Women’s Perceptions on Newborn Care Practices, Knowledge Sources, Benefits, and Challenges in Rural Northern Jordan: A Qualitative Study

**DOI:** 10.3390/healthcare14010052

**Published:** 2025-12-24

**Authors:** Mahmoud H. Alrabab’a, Roqia S. Maabreh, Dalal B. Yehia, Anwar M. Eyadat, Abdallah Ashour, Salam Bani Hani, Amira A. Mohammad, Naser A. Alsharairi, Yazan Alkhsealat, Hanan Abusbaitan, Wael T. Alali

**Affiliations:** 1 Prince Al Hussein Bin Abdullah II Academy for Civil Protection, Al-Balqa’ Applied University, Amman 11183, Jordan; alkhsealat.yazan343@yahoo.com (Y.A.); wael.t.alali@gmail.com (W.T.A.); 2 Nursing College, Irbid National University, Irbid 21110, Jordan; roqiamaabreh45@gmail.com (R.S.M.); a.abuashour@inu.edu.jo (A.A.); s.banihani@inu.edu.jo (S.B.H.); 3 Faculty of Nursing, ISRA University, Amman 11622, Jordan; bashir_dalal@yahoo.com; 4 Faculty of Nursing, Hashemite University, Zarka 13133, Jordan; anwareyadat@yahoo.com; 5 Faculty of Nursing, Al-Ahliyya Amman University, Amman 19328, Jordan; 10615@ammanu.edu.jo; 6 Heart, Mind and Body Research Group, Griffith University, Gold Coast, QLD 4222, Australia; naser.alsharairi@gmail.com; 7 Horizon for Research & Studies, Irbid 21110, Jordan; 8 School of Nursing, Oakland University, Rochester, MI 48309, USA; habusbaitan@oakland.edu

**Keywords:** Jordanian women, newborn care practices, qualitative, cultural practices

## Abstract

**Background/Aim**: Communities all across the world celebrate the birth of babies through distinct customs and traditional practices. While some of these traditions may bring comfort and cultural continuity, others may not be in line with medical recommendations and could pose major health risks to the newborn. This study examined rural Jordanian women’s perceptions on practices, knowledge sources, benefits, and challenges around caring for newborns in the northern region. **Materials and Methods**: In this qualitative descriptive study design, twelve women (aged 22 to 60 years) from the Kufr Som village in Northern Jordan, took part in in-depth semi-structured interviews in August 2025. The interviews focused on identifying caregiving practices, knowledge sources, and perceived benefits or challenges related to newborn care. The responses were verbatim transcribed from audio recordings for thematic analysis. **Results**: Nine themes emerged. “Thermal protection,” “bathing care,” “umbilical cord care,” and “feeding rites” are four themes that encapsulate the common practices women follow when caring for a newborn. The two themes that capture the sources of knowledge and direction for learning newborn care practices are “transmission of knowledge across generations” and “social influence”. The themes “spiritual safeguarding” and “perceived health protection” highlight the benefits of traditional practices, whereas “conflicts between tradition and modern care” underscores their challenges. **Conclusions**: Newborn care practices are deeply rooted in Northern Jordanian culture. Evidence-based strategies are needed to augment existing practices in order to improve neonatal care outcomes.

## 1. Introduction

Neonatal survival and the best possible care for newborns are emphasized by the World Health Organization (WHO) as top healthcare priority [1]. Cultural traditions continue to influence early newborn practices in many communities around the world, despite a constant need for standard improvements in neonatal care [2,3,4]. The WHO recommends newborn care practices, including breastfeeding, umbilical cord care, and thermal protection. Women should receive support for exclusive breastfeeding from birth until six months of age. Infants should wear clothing suited to environmental conditions, with the inclusion of a hat and one or two layers as appropriate. For neonates delivered at home in environments with a high neonatal mortality rate, daily administration of chlorhexidine to the umbilical cord stump during the first week of life is recommended [1]. Newborn care practices are influenced by a number of factors, such as the availability of maternity care, the prevalence of infections, socioeconomic status, healthcare infrastructure, traditional care providers, and cultural and religious traditions [4,5].

Aside from WHO recommendations, neonatal care practices vary greatly between countries. For example, refraining from showering during the first month after giving birth, encouraging hot foods while avoiding cold ones, and having mystical healers conduct rituals and blessings are the most common traditional practices in Asian countries [2].

Women in sub-Saharan African countries keep newborns warm, clean them shortly after delivery to get rid of blood and grime, and use substances on the cord to hasten its removal [3]. In Africa, the use of methylated spirit, hot compresses, sand mixed with saliva, herbal preparations, and ashes in umbilical cord care carry particular clinical risks and are linked to increased infections [4,6].

Women from various ethnic groups in Turkey followed distinct traditional practices. For instance, Arabs used anise tea to ease gas and olive oil to treat diaper rash, Kurdish preferred fennel tea, while Assyrians put salt to the umbilical cord [7]. Turkish postpartum women engage in practices such as bathing the newborn’s head with a lead cup to ward off evil spirits and placing the Quran under the mother’s pillow [8]. Egyptian postpartum practices typically bathe neonates after delivery, apply substances to the neonatal umbilical stump, and clean the eyes with breast milk [9].

In rural Jordanian communities, traditional newborn care practices include restrictive swaddling, rubbing salt and olive oil on the newborn’s skin, using kohl on the newborn’s eyes, covering the newborn’s umbilical stump with coffee grounds or kohl powder, applying a mixture of flour, egg yolk, onion to the baby’s stomach, and performing ritual bathing [10,11,12,13]. Other practices include giving newborns honey, fenugreek (a herbaceous plant of the pea family), and anise at the initial feeding, treating minor respiratory conditions with a mixture of lemon and honey, olive oil, and sesame oil, and treating jaundice by giving newborns water with sugar, or “dextrose water” [12,13].

Adequate knowledge and newborn care practices allow caregivers to be more aware of warning signs and unsafe traditional practices [14]. Mothers often adopt practices from their mothers-in-law, grandmothers, and neighbors, establishing social norms that may undermine adherence to clinical practices [12]. Women in rural communities often express uncertainty about traditional and clinical practices for newborn care, a phenomenon documented in Jordan and other low- and middle-income countries [15,16]. However, examining the cultural context is significant to improving newborn care, given the potential health risks associated with traditional practices.

The aforementioned studies indicate that newborn care practices in rural Jordan are notably different from those in other countries. To date, no qualitative study has explored how women in the northern rural areas of Irbid city perceive newborn care practices, knowledge sources, and related benefits and challenges. Understanding neonatal care practices is crucial, particularly in rural areas where family and community norms heavily influence care decisions. This would facilitate the development of evidence-based strategies that align traditional practices with standard clinical guidelines. Thus, this qualitative study could provide valuable insight into how women in Northern Jordan navigate between traditional beliefs and medical advice, information that is vital for encouraging culturally responsive health practices.

## 2. Methods

### 2.1. Research Design

A qualitative research design was used to explore the practices, sources of knowledge, benefits, and challenges related to caring for newborns in the northern region of Jordan. The research methodology was prepared in accordance with the Consolidated Standards for Reporting Qualitative Research (COREQ).

### 2.2. Participants and Setting

The research was carried out in Kufr Som, which is located in Bani Kinanah region of the Irbid Governorate in Northern Jordan. This village was selected to reflect the critical role of women in newborn care and the difficulties they face in accessing modern healthcare services [17].

Women selection was based on the following criteria: (1) resided in Kufr Som and were at least 18 years old; (2) had delivered a baby within the last 12 months; (3) were mothers-in-law or older female relatives involved in newborn care; and (4) were able to freely express their opinions on newborn care practices. Women were recruited using purposive and snowball sampling methods in order to find those who had a wealth of experience and familiarity with traditions. Data saturation was assessed to make sure that enough information had been collected. After the first and second authors (MA and RM) completed the sample selection, the responses of twelve women were reviewed and reported. Saturation was monitored throughout data collection and analysis, with no new codes or themes emerged after the twelfth interview. Interviews were conducted in the women’s homes after informed consent was obtained. To safeguard their identities, each women was assigned a distinct code, labeled WP1 through WP12.

### 2.3. Data Collection

Semi-structured in-depth interviews were used to gather data, and women provided specific personal experiences and practices based on their own knowledge. Three main questions guided the interviews (Supplementary Table S1): (1) What traditions or normal practices do mothers in your society adhere to when taking care of a newborn? (2) Where did you learn these practices, and who typically helps or advises new mothers on how to care for their newborn? and (3) In your opinion, what are the benefits or challenges of traditional practices? Additionally, demographic details such as age, marital status, parity, educational background, and role in caring for newborns were documented. Following the initial development of the interview guide by the first two authors, the remaining authors (DY, AE, AA, SB, and AM) participated in improving the flow of the questions.

The interviews, which took place in women’s homes throughout August 2025 and lasted between 30 and 60 min, were conducted in Arabic, which the participants felt was the most comfortable language.

### 2.4. Analyzing Data

The Braun and Clarke thematic analysis approach, which involves script reading, code development, and theme identification, was used to analyze the data [18]. The coding process was carried out manually. The authors carefully reviewed each tape multiple times and made preliminary descriptive and conceptual notes in order to gain a comprehensive grasp of women’s perceptions regarding the care of newborns. Following a full transcription of the responses, the transcripts were cleaned up and edited in order to identify and organize codes. Codes were divided into broad topic groups in order to identify patterns of meaning. The codes were converted into sub-themes by additional data analysis, and they were subsequently incorporated into the main themes.

### 2.5. Research Team

The authors (MA, YA, and WA) are current faculty members and experts in the data gathering, analysis, and design of maternity, newborn, and adult nursing projects. The authors (RM, DY, AE, AA, SB, AM, and HA) are all active faculty members with a wealth of experience in community health nursing and qualitative data analysis. The author (NA) is a public health researcher with expertise in data gathering and analysis.

### 2.6. Reflexivity and Trustworthiness

The researchers employed a number of strategies to ensure the method was reliable and credible. The shared cultural background of the authors (MA and RM) as women enhanced rapport during the interviews. The same authors were also contributed to developing participant summaries of the findings as part of the member checking process. The authors (DY, AE, and AA) carried out a comprehensive analysis to understand the disclosures made by women and generate initial codes. Regular collaborative discussions with the other authors (AM, NA, YA, HA, and WA) were conducted to resolve discrepancies in interpretation and to reduce potential bias, thereby enhancing the rigor of the final coding and the study’s credibility.

The translation of the transcripts from Arabic to English was performed by the first two authors, and the translations were subsequently verified by other authors (DY, AE, AA, SB, AM, YA, and WA) to confirm accuracy of meaning. Back-translation from English to Arabic was conducted by two authors (NA and HA), who have advanced knowledge of Arabic to ensure meaning equivalence, thereby improving rigor and reducing potential bias in coding and thematic analysis.

### 2.7. Ethical Approval

Irbid National University’s Faculty of Nursing Research Ethics Committee granted ethical permission (Ref: IRB0018). Consent was requested prior to the interview because participation was completely optional. To preserve the participants’ privacy and anonymity, no personally identifying information was recorded.

## 3. Results

### 3.1. Demographic Characteristics

The demographic details of the twelve women are listed in Table 1. The age range was 22 to 60 years old, with six women had recently given birth, while the remaining were older women and mothers-in-law. All of the women were married, and just one was widowed. Two women had no formal education, three had degrees from universities, and seven had only completed elementary or secondary schooling. The number of births varies from one (parity 1) to eight (parity 8).

### 3.2. Key Thematic Findings

A wide range of newborn care practices were identified. The three guiding interview questions—traditional newborn care practices, knowledge and guidance sources, and the benefits and challenges of traditional practices—are used to arrange the thematic findings (Table 2). An audit trail that includes data synthesis through the extraction and abstraction of findings in common themes and subthemes was shown in Supplementary Table S2.

#### 3.2.1. Traditional Practices of Newborn Care

**Thermal protection practices**

Women stressed the importance of keeping the room warm and wrapping the infant firmly in warm clothing. Newborns lack the ability to control body temperature, so keeping them warm is vital. This helps conserve oxygen and energy, which are critical for growth and development.

WP1 shared “*That day, my son, who was just a few months old, would not stop weeping...and I have no idea why...I shut the window and gave him a solid warmth...Then he became serene and drowsy*”.

WP8 expressed, “*If the babies are exposed to cold air, they will get sick quickly from the bad weather…so we have to cover them tightly well in a warm cloth for at least three months*”.

**Umbilical cord practices**

Women reported using olive oil, salt, and herbal powder to heal the cord. Although these practices maintain the stump dry and tidy, they might facilitate bacterial exposure, thereby increasing the risk of infection.

WP6 shared “*My mother asked that I used herbal powder to hasten the baby’s cord separation...I simply diluted it with water since I was worried that it may injure my daughter*”.

WP8 mentioned “*In our tradition...we use olive oil to separate cords with children...God bless my children…they feel well after this*”.

**Bathing practices**

Women mentioned delaying the newborn’s bathing so they could utilize oil massages or herbal medicines later. Although these practices may be culturally comforting, they may cause skin discomfort for newborns.

WP9 explained “*We don’t bath the baby immediately, until a couple of days...We wait and then wash with herbs and olive oil*”.

**Feeding practices**

Women reported giving their newborns sugar water and anise tea before breastfeeding, believing that these practices would prevent stomachaches.

One women stated “*My mother said that anise tea soothes the stomach…So she gave it to my baby before I started breastfeeding*” (WP4).

WP6 shared “*My mother began giving my son sugar water in the first few days following his birth...She said that it keeps them from being constipated*”.

#### 3.2.2. Sources of Knowledge and Guidance

**Transmission of knowledge across generations**

Women credit their mothers for giving them newborn care practices, highlighting the generational transfer of knowledge as a major theme. Older women were also highly valued in guiding newborn care. Younger mothers generally felt obliged to follow their instructions. Involving elders in newborn care provide emotional support to new mothers and sustain cultural continuity.

WP7 reported “*Having [my mother] around made me feel happy...When I gave birth, all I needed was someone to look after my child...My mother merely kept telling me to breastfed my child on a regular basis*”.

WP10 shared “*She [my mother] decides how the baby should be cared for…I usually follow her instructions carefully*”.

**Social influence**

Women indicated that friends and neighbors play a social role in shaping their newborn care practices. Friends and neighbors provide mothers with practical and emotional assistance, with their influence being significant in communities that respect traditional knowledge.

WP9 expressed “*I have to carefully and unquestioningly follow the advice of the older women [my neighbor]…Her knowledge of newborn care is extensive*”.

WP10 said “*I simply felt that I had to do as she [my friend] said...If she hadn’t told me everything there was to know about baby care...I would never have learned all of these things*”.

#### 3.2.3. Benefits and Challenges of Traditional Practices

**Belief in health protection**

Women believed that using thermal protection, bathing, and feeding practices kept babies healthy and protect them from disease. Integrating medical guidance with traditional practices is important to maintain newborns’ well-being.

WP9 shared “*We firmly believe that keeping babies warm by covering them with extra clothing often shields them against disease and other evil outcome*”.

WP11 expressed, “*Our practices have kept children healthy for generations…The baby grows healthily when we massage them with olive oil*”.

**Spiritual safeguarding, cultural continuity**

Women explained that religious rites were necessary to protect the baby from bad spirits. The most common practices included reciting Quran, whispering the adhan to the newborn, and applying protective charms. These believed to protect the baby from spiritual harm.

WP11 said “*Right after birth...we whisper the adhan in the baby’s ear and recite Quran to protect them from any evil outcomes and ‘bad eyes’*”.

**Conflict between traditions and medical care**

Women expressed perplexity when they encountered conflicting recommendations and sporadic criticism from medical professionals. Their choice to choose what is best for the baby was restricted by family pressure to follow traditions. Certain traditional practices of caring for newborns may put their health at danger or cause delays in receiving medical care if they go against evidence-based recommendations.

WP1 shared “*He [the doctor] told me not to eat too much spicy food while pregnant because it could burn my stomach and harm my unborn child. I don’t like spicy food because of this, but my mother insists that I eat it even though she keeps making meals with a lot of chili powder*”.

WP4 stated “*Sometimes I get confused on who to follow since the doctor says one thing and my mother says another...so I find myself unable to decide on who to follow*”.

## 4. Discussion

This study is the first to examine how rural Jordanian mothers in the northern region care for their newborns. Nine themes were identified and categorized into three main topics: (1) traditional newborn care practices, (2) knowledge and guidance sources, and (3) benefits and challenges of traditional practices.

### 4.1. Traditional Newborn Care Practices

Northern Jordanian culture has a strong tradition of women caring for their newborns. The key findings demonstrated that thermal protection, umbilical cord, bathing, and feeding customs are among the noteworthy practices. Recently delivered women, older women, and mother-in-law exhibited thermal protection practices, such as swaddling babies warmly to prevent exposure to the cold. This practice is not unique to Jordan; qualitative research conducted in Nigeria and India has emphasized the significance of wrapping babies in clothing to protect them from cold [19,20,21]. Al-Sagarat & Al-Kharabsheh [11] suggested that wrapping babies with qmat keeps them warm throughout the winter in a prior qualitative study conducted in four rural areas of Amman, the capital city of Jordan. These studies underscore the importance of maintaining neonatal warmth and comfort while deepening our understanding of the ways cultural values shape maternal behaviors in Northern Jordan.

Women who had recently given birth and older women in this study healed their umbilical cords with herbal medicines, salt, and olive oil. Prior qualitative studies have documented umbilical cord practices among Jordanian rural women, including the use of alcohol, antibiotics, neomycin powder, and breast milk [10,11,12]. Numerous qualitative research also showed that women in many different cultures used a variety of umbilical cord practices. For example, Indian women use sweet flag, coconut oil, cow dung, ashes, and powdered turmeric, face, and talc [19,20,22,23,24]; Nigerian women use shea butter, chimney powder, methylated spirit, cooking pepper, alligator pepper, ash, toothpaste, and saccharin [21,25]; Ghanaian women use shea butter [26]; Ugandan women use dust [27]; Pakistani women use mustard oil with salt or turmeric [28]; Bangladeshi women use boric powder and mustard oil [29]; and Moroccan women use henna powder with kohl [30]. Tanzanian women tie the umbilical cord around the neck to keep the baby’s navel from landing on his or her genitalia [31]. Unsafe umbilical cord practices in rural communities such as using cow dung, sand, or salt with saliva can pose a risk of infections [6]. The diversity and cultural significance of umbilical cord care practices are highlighted by these comparisons, illustrating that using salt, olive oil, and herbal remedies did not align with those observed worldwide. Comparing these practices with those in other countries can gain insights into local beliefs, create safe newborn care interventions, and implement measures to reduce harmful practices.

In this study, mothers-in-law, older women, and women who had just given birth frequently delayed bathing their newborns in order to give them oil massages or herbal medicines later. These findings are somewhat in line with earlier qualitative studies carried out in rural Jordan, which found that the mother, mother-in-law, and female family members assist with bathing the newborn by massaging them with olive oil, adding salt to the bath water, and washing them right away with water and soap to remove blood [10,11,12,13]. Newborn bathing practices differ by countries. For instance, it was traditional in India to massage the infant with olive oil, castor oil, coconut oil, turmeric-infused oils, turmeric paste, ragi flour, dhal, and cumin water [19,20,22,23,24]. In Bangladesh, Nigeria, and Morocco, newborns are bathed with warm water alongside silver and gold ornaments [21,29,30]; in Ghana, herbal baths are customary [32]; in Pakistan, mustard oil is applied during bathing [28]; and in Turkey, babies are bathed using mixtures of salt and olive oil [33]. These comparisons demonstrate that newborn bathing practices in Northern Jordan align with a global pattern, with local practices impacting the timing, techniques, and materials utilized in newborn care. Highlighting the influence of family elders and traditional knowledge on newborn care enables a clear understanding of how Northern Jordanian practices fit within regional and global contexts. Such insights can inform culturally appropriate health interventions that support bathing practices and enhance newborns health outcomes.

A mother who recently gave birth and mother-in-law reported giving their babies sugar water or anise tea before breastfeeding. Qualitative research in rural Jordan reported that mothers and mothers-in-law provide warm water in feeding bottles to newborns along with anise, chamomile, herbal tea, black tea, fenugreek, and formula milk [10,12,13]. Qualitative studies indicate that in China, Bangladesh, India, and Turkey, neonates are commonly given sugar water, honey, castor oil, jaggery powder, and honeysuckle herb shortly after birth by mothers, mothers-in-law, and older women [19,20,22,24,29,33,34,35]. This study indicates that early feeding practices in Northern Jordan are shaped by regional traditions. Understanding discrepancies among studies shed light on how women and mothers-in-law in Northern Jordan give sugar water or anise tea prior to breastfeeding, highlighting the influence of cultural beliefs on neonatal feeding practices. This insight broadens our understanding of the social and cultural dimensions that shape neonatal care in Jordan.

### 4.2. Knowledge and Guidance Sources

The study examining the sources of knowledge and guidance behind newborn care practices identified two main influences: intergenerational transmission and social interactions. Women reported that their mothers, especially those who were older, taught them the newborn care practices. Older women are regarded as having greater knowledge that could influence the care of newborns. The current analysis is in line with a number of qualitative studies. For instance, Bangladeshi women avoided certain foods, including sour fruits and peppers, based on recommendations from elder female relatives [29]. Women in Turkey and Morocco seek guidance from family elders, particularly mothers-in-law, on how to feed their newborns [30,33]. Pakistani women depended heavily on extended relatives, particularly paternal grandmothers, for guidance in caring for their babies [28]. Chinese women adopt newborn care practices guided by the experiences of their relatives, usually their grandmothers or grandmothers-in-law [35]. Ethiopian women are taught by elder family members how to massage their newborns [36]. According to a study by Mrayan et al. [10], elder female relatives serve as the primary sources of knowledge in Jordanian settings. These comparisons emphasize the significance of intergenerational knowledge by demonstrating how elder female relatives are crucial in influencing newborn care practices across many cultures. This study enhances understanding of how comparable patterns operate in Northern Jordan, where older women continue to be influential in maintaining caregiving practices. This aids in elucidating the cultural roots that still shape newborn care in Jordanian households.

Some women noted that their neighbors and peers provided recommendations regarding newborn care practices. According to Arabiat et al. [12], group norms among Jordanian mothers influence the practice of newborn care. Women in Turkey obtain guidance on feeding and caring for their newborns from neighbors [33]. Ghanaian mothers receive spontaneous counsel from relatives and neighbors when their babies are sick [32]. This study illustrates how social networks impact newborns care, indicating that Jordanian mothers’ dependence on peers and neighbors is part of a broader cultural pattern. The study enhances knowledge of how social networks influence caregiving decisions in Northern Jordan by comparing the results with regional and global data. These findings highlight the need to incorporate local social contexts into interventions intended to improve newborn health.

### 4.3. Benefits and Challenges of Traditional Practices

According to the study, mothers believed that thermal protection (keeping the baby warm), bathing (massaging with olive oil), and feeding (using sugar water/anise tea) practices preserved the health of newborns and avoided them from disease. A Jordanian study found that swaddling reduces colic and respiratory conditions and is believed to promote straight leg development in babies [10]. Other local research found that wrapping and tying children may improve sleep duration by preventing disturbances from unexpected hand movements [11]. Evidence from Ethiopia indicates that delaying bathing may reduce the risk of hypothermia [37]. Comparing findings from Jordan with evidence from other countries, the study enhances understanding of why traditional practices persist and how they are viewed as beneficial for infant health. Growing awareness of the benefits of using traditional practices in Northern Jordan could help shape newborn healthcare policies.

Spiritual protection and cultural continuity are two further advantages that women reported while using traditional practices for newborn care. This study identifies the recitation of the adhan to newborns as a traditional practice. Women stated that the rituals serve as spiritual defense against the evil eye. According to other research, the reasons behind this protective cultural practice are well understood in rural Jordan [11,12]. This demonstrates how rural Jordanian culture relies on well-known Arabic beliefs to protect newborns. Qualitative research from other countries also supports the use of this practice. Moroccan women believed that the recitation of the Quran serves to protect newborn from the evil eye [30]. Bangladeshi mothers reported placing a small matchbox near the baby’s head along with a few pieces of cow bone as a protective measure against evil spirit [29]. Indian mothers recited a ceremonial mantra to protect the babies from malevolent diseases [19]. Indian women also believed that performing certain rituals, wearing black bangles, tying black thread to the hand or leg, and keeping bindhi on the forehead, cheek, and foot using kajal or burnt vasambu could help against the evil eyes [24]. These findings contribute to a better understanding how spiritual and protective rituals are in newborn care across numerous communities. The study emphasizes that Jordanian beliefs regarding spiritual protection and the evil eye are intertwined with broader cultural norms that impact maternal practices. This highlights the importance of considering these practices when analyzing maternal behavior and creating culturally appropriate health interventions in Northern Jordan.

This study found that regarding feeding practices (such consuming spicy food), women encounter conflicting advice and intermittent criticism from medical professionals. Family pressure to follow traditional customs constrained their ability to make the best decision for their newborn. A research carried out in rural Jordan found that although mothers who visit clinics receive medical guidance, they often continue following traditional customs out of respect or concern for the disapproval of their families [10]. Jordanian mothers frequently follow the guidance of their grandparents or mothers-in-law, even if they hold different opinions [11]. Other qualitative research also addressed the conflict between tradition and modern care. Indian women claimed that babies died from jaundice as a result of the village elders discouraged hospitalization, insisting it was unnecessary [23]. Other Indian women followed the guidance of doctors and nurses, as many hospitals have become accessible [24]. Ghanaian women reported that they were unable to prevent mothers or mothers-in-law from giving care that contradicted against medical advice [32]. Ethiopian women reported how they are foregoing customs since they are receiving guidance and help from medical professionals [36]. This study highlights the continuous struggle mothers encounter between adhering to medical advice and traditional expectations. The study expands knowledge of how family involvement, cultural norms, and evolving access to healthcare shape newborn care decisions in Jordan. These insights shed light on the reasons behind the persistence of traditional practices in Northern Jordan and suggest strategies to align healthcare message closely with mothers’ real-world experiences.

## 5. Study Limitations

This study may be constrained by the representativeness of a small sample size and a single rural area. Response bias may arise from the use of self-reported data and women’s recollection of past practices. In addition, the ethnic, cultural, and religious diversity of Northern Jordan implies that certain traditions may differ across specific subgroups.

## 6. Conclusions and Recommendations

This qualitative study identified nine themes that represented the practices, knowledge sources, benefits, and challenges around caring for newborns in rural Northern Jordan. A number of newborn care practices, such as thermal protection, bathing care, umbilical cord care, and feeding rituals, were reported by recently delivered mothers, older women, and mother-in-law. Elderly mothers and members of the social community, such as peers and neighbors, are the main sources of knowledge and guidance for women regarding newborn care practices. Women believed that traditional practices improved and preserved the health of babies. Women identified two benefits of using traditional newborn care practices: spiritual safeguarding and cultural continuity. Women described confusion due to inconsistent guidance and sporadic criticism from medical professionals regarding traditional newborn care practices.

Engaging older women and family members in community education initiatives may enhance wider adoption of safe practices. Neonatal health outcomes may be strengthen by supporting traditional practices that align with evidence-based clinical safety while eliminating detrimental ones. The results highlight that healthcare providers in rural Northern Jordan should adopt culturally sensitive strategies in newborn care promotion. Increasing clinicians’ awareness of newborn care practices would allow them to recognize women’s views and experiences while explaining evidence-based recommendations. Such training may alleviate the uncertainty and frustration women expressed when received contradictory advice or criticism from health experts. From a policy perspective, closing the gap between medical guidance and women’s lived experiences would be more achievable through active community engagement and continuous supervision.

## Figures and Tables

**Table 1:** Sample characteristics.

Participants ID	Age (Years)	Education Level	Marital Status	Parity (No. of Children)	Role in Newborn Care
WP1	26	Secondary	Married	1	Recently delivered mother
WP2	34	Primary	Married	3	Recently delivered mother
WP3	29	University	Married	2	Recently delivered mother
WP4	38	Secondary	Married	4	Recently delivered mother
WP5	22	No formal education	Married	1	Recently delivered mother
WP6	31	University	Married	2	Recently delivered mother
WP7	47	Secondary	Married	6	Mother-in-law
WP8	55	Primary	Married	7	Older women
WP9	60	No formal education	Widowed	8	Older women
WP10	42	University	Married	5	Mother-in-law
WP11	53	Primary	Married	6	Older women
WP12	45	Secondary	Married	5	Mother-in-law

**Table 2:** Data reduction and thematic outcomes.

Interview Question/Prompt	Subthemes	Themes
What traditions or normal practices do mothers in your society adhere to when taking care of a newborn?	Keeping baby warm	Thermal protection practices
	Cord treatment	Umbilical cord practices
	Bathing customs	Bathing practices
	Feeding rituals	Feeding practices
Where did you learn these practices, and who typically helps or advises new mothers on how to care for their newborn?	Learning from mother Older women’s decision-making	Transmission of knowledge across generations
	Community advice	Social influence
In your opinion, what are the benefits or challenges of traditional practices?	Physical benefit	Belief in health protection
	Spiritual benefit	Spiritual safeguarding, cultural continuity
	Conflicting advice	Conflict between tradition and medical care

## Data Availability

The study’s data are not publicly accessible due to ethical restrictions and participant privacy and confidentiality.

## Supplementary Materials

The following supporting information can be downloaded at https://www.mdpi.com/article/10.3390/healthcare14010052/s1. Table S1. Interview guide; Table S2. Results abstraction and extraction.
