# Supplementary material for: Women’s Perceptions on Newborn Care Practices, Knowledge Sources, Benefits, and Challenges in Rural Northern Jordan: A Qualitative Study
Source: Healthcare (Basel). 2025 Dec 24;14(1):52. doi: 10.3390/healthcare14010052 (PMC12785348; doi:10.3390/healthcare14010052)
Supplement: Supplementary file 1 [file healthcare-14-00052-s001.zip › healthcare-4020902-supplementary.pdf]

Supplementary Table S1. Interview guide.

Topic of interview	Question	Aim of the question
Cultural practices in newborn care	What traditions or practices do women follow in your community when caring for a newborn?	Explore cultural traditions and caregiving practices
Sources of knowledge and support in newborn care	Where did you learn these practices, and who usually gives new moms advice or assistance on how to take care of their baby?	Identify the sources of knowledge and guidance
Traditional newborn care practices	What are the benefits or challenges of traditional practices, in your opinion?	Identify benefits and drawbacks
Participant background	What is your age?<br>How many children do you have?<br>Are you married or not?<br>What is your highest level of education?<br>Could you describe your role in caring for newborns?	Participant characteristics

Supplementary Table S2. Results abstraction and extraction.

Themes	Subthemes	Codes	Quotations extracted
Thermal protection practices	Keeping baby warm	Closing windows to prevent drafts<br><br>Keeping the room warm<br><br>Layering baby's clothing	*"I was told to keep the room warm all the time so that my baby doesn't catch cold"* (WP12)
Umbilical cord practices	Cord treatment	Using oils<br><br>Using salt<br><br>Using herbal powder	*"My mother told me put olive oil or sometimes salt on the cord to help it dry quickly"* (WP2)
Bathing practices	Bathing customs	Postponed first bath<br><br>Water bathing<br><br>Olive oil bathing<br><br>Herbs bathing	*"After my four children were born, I applied herbal remedies to their skin...and she [my sister] did the same for their children"* (WP4)<br><br>*"I used olive oil to massage all of my kids...It offers them more strength and softens their skin...I did that every day"* (WP10)<br><br>*"We never wash the babies right after birth…No. Instead, we do so only after a few days, whereby we use special herbs with water…then rub olive oil on his skin…[we believe that] it makes them strong"* (WP11)
Feeding practices	Feeding rituals	Using sugar water<br><br>Using anise tea	*"My mother told me to give a spoon of sugar water…She said it clears the baby's stomach and keep it free from infections"* (WP3)<br><br>*"I informed my daughter that a baby's stomach would be cleared if they were given water with sugar...My daughter fed her son with a spoon since he*

			refused to drink from the bottle" (WP12)
Transmission of knowledge across generations	Learning from mother<br><br>Older women's decision-making	Maternal guidance as primary source<br><br>Obligation to follow elders' mother instructions	*"She [my mother] decides everything...Even if I want to do what the doctor said...I cannot go against her"* (WP4)<br><br>*"Everything I know, I learned from my mother...She raised many children and she knows best"* (WP5)<br><br>*"That was in the summer...She [my mother] told me to eat fish while pregnant because your body is hot"* (WP6)<br><br>*"My mother taught me everything about caring for my baby...I follow what she instructed me to do and what she did showed me"* (WP12)
Social influence	Community advice	Advice from neighbor<br><br>Advice from friend	*"When we gather for any meal, for example tea, the older women [my friend] always remind us what is good for the baby...They all share their experience"* (WP2)
Belief in health protection	Physical benefit	Disease prevention<br><br>Perceived bodily health	*"Giving babies herbal tea frequently after birth has kept their stomachs healthy and prevented constipation"* (WP12)
Spiritual safeguarding, cultural continuity	Spiritual benefit	Quran recitation<br><br>Practices to ward off evil or harm	*"Reciting Quran keeps away jealousy and brings blessings to the baby… and it truly connects us with our faith and our mothers' traditions"* (WP7)<br><br>*"The very first thing we do for these babies is to recite the adhan in their ears...Without that, we cannot guarantee that the child is not truly protected"* (WP8)

			"Quran keeps away the evil eye and brings blessings to the baby…it is much better than the hospital ways" (WP9)
Conflict between tradition and medical care	Conflicting advice	Contradictory guidance<br><br>Advice overload	*"He [the doctor] told me not to give herbs...but if I refuse...my family will say I don't respect our traditions"* (WP3)<br><br>*"She [the nurse] told me to breastfeed right away, but my mother insisted on giving anise tea first…Since it was hard to decide which way to go, so I tried to do both, just to keep everyone happy"* (WP5)<br><br>*"The nurse at the health center told me to breastfeed immediately…but I still gave sugar water first"* (WP6)
